# A population-based cohort of adults with asthma: mortality and participation in a long-term follow-up

**DOI:** 10.1080/20018525.2017.1334508

**Published:** 2017-06-16

**Authors:** Helena Backman, Linnea Hedman, Caroline Stridsman, Sven-Arne Jansson, Anne Lindberg, Bo Lundbäck, Eva Rönmark

**Affiliations:** ^a^ Department of Public Health and Clinical Medicine, Division of Occupational and Environmental Medicine/the OLIN Unit, Umeå University, Umeå, Sweden; ^b^ Department of Health Sciences, Luleå University, Luleå, Sweden; ^c^ Department of Public Health and Clinical Medicine, Division of Medicine/the OLIN Unit, Umeå University, Umeå, Sweden; ^d^ Krefting Research Centre, Institute of Medicine, University of Gothenburg, Gothenburg, Sweden

**Keywords:** Public health, risk factors, natural history, obesity, ischemic heart disease, socioeconomic status

## Abstract

**Background and objective**: Asthma is a major public health concern. The aim of this study was to characterize a large population-based cohort of adults with asthma, and to study factors associated with all-cause mortality and non-participation in a long-term follow-up.

**Design**: Random and stratified samples from five population-based cohorts were clinically examined during 1986–2001, and all subjects with asthma were included in the study (*n* = 2055, age 19–72 years, 55% women). Independent associations between different risk factors and (i) mortality and (ii) non-participation in a clinical follow-up in 2012–2014 were estimated.

**Results**: In 1986–2001, 95% reported any wheeze and/or attacks of shortness of breath in the past 12 months, and/or asthma medication use. Over the up to 28 years of follow-up time, the cumulative mortality was 22.7%. Male gender, current smoking, and lower forced expiratory volume in 1 sec of predicted (FEV_1_% of predicted) were independent risk factors for mortality, while obesity was associated with non-participation in the follow-up. Older ages, ischemic heart disease, and low socioeconomic status were associated with both mortality and non-participation.

**Conclusions**: The risk factors associated with mortality in this adult population-based asthma cohort were similar to those commonly identified in general population samples, i.e. male gender, current smoking, and lower FEV_1_% of predicted, while obesity was associated with non-participation in a long-term follow-up. Ischemic heart disease, low socioeconomic status, and older ages were associated with both mortality and non-participation.

## Introduction

Asthma is a major public health concern which places a considerable burden on society in terms of morbidity, mortality, and costs [1]. It is a common disease of differing severity presenting with several phenotypes [2]. Non-allergic childhood asthma often remits, while the majority of allergic childhood asthma persists into adulthood [3–6]. In contrast to childhood asthma, adult-onset asthma is often more persistent and non-atopic [7–9]. Despite a large number of studies on asthma, our ability to predict persistence, remission, or mortality is limited [10].

Subjects with asthma have long been reported to have excess all-cause mortality compared to subjects without asthma [11–13], although the excess mortality among subjects with asthma seems to be declining, according to recent studies [14–17]. This excess mortality risk is related to lower pre-bronchodilator forced expiratory volume in 1 sec (FEV_1_) [12,18–21] and large FEV_1_ bronchodilation response [22], but studies presenting factors associated with mortality among subjects with asthma are scarce.

Both the diagnosis and therapeutic management of asthma have changed during the past few decades, and asthma is more frequently diagnosed today than during the 1980s and 1990s [23,24]. Furthermore, general knowledge about asthma has increased in the community. Thus, there are likely to be differences over time in what a self-reported physician diagnosis in epidemiological studies represents. Therefore, not only diagnosis but also other factors such as respiratory symptoms, lung function, bronchial hyperreactivity, and medication use should be taken into account [24]. The participation rates in epidemiological studies have declined over time [25,26], but whether and how this affects the results remains to be determined.

Long-term follow-ups of asthma cohorts enable studies on factors related to persistence, remission, relapse, and progression of the disease. As we still do not know how to prevent asthma, increased knowledge on factors related to disease progression can contribute significantly to improved public health. This knowledge is especially limited concerning adults. While patient-based asthma cohorts are more likely to include subjects with moderate and severe disease, population-based asthma cohorts represent the entire asthma population in a society. However, few well-characterized population-based asthma cohorts have been studied over the long term, although such studies are warranted [8].

The aim of this study was to characterize a large cohort of adults with asthma identified by clinical examinations of population-based samples in northern Sweden during 1986–2001. A further aim was to study factors associated with all-cause mortality and non-participation in a long-term follow-up of this population-based asthma cohort.

## Material and methods

### Study area

The study was performed in Norrbotten, the northernmost county of Sweden, with a population of about 250,000 inhabitants. The climate is subarctic, with long winters and short but warm summers. The study was performed as a part of the epidemiological research program the Obstructive Lung Disease in Northern Sweden (OLIN) studies and was approved by the Regional Ethical Review Board at Umeå University.

### Study sample

**Figure 1.**
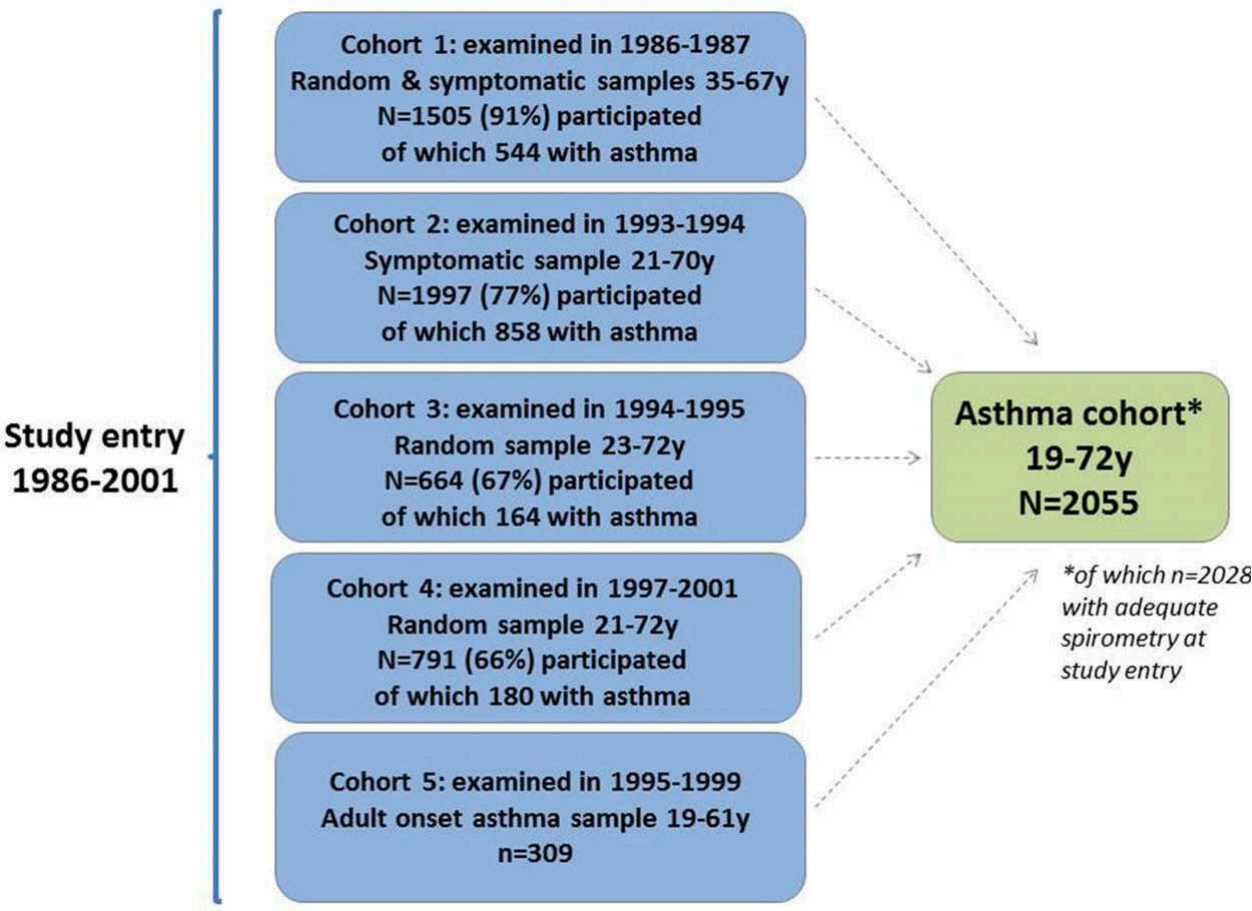
Summary of the entire cohorts from where the asthma cohort was derived. The asthmatics were identified in clinical examinations of random and stratified samples of the cohorts.

The study sample consists of a large cohort of adults with asthma (*n* = 2055) (Figure 1) which was identified in clinical examinations of five previously described population-based cohorts within the OLIN studies. Informed consent was obtained from all individual participants included in the study. Initially, cohort 1 was an age-stratified total population sample recruited in 1985 (*n* = 5697; 86% of invited; 35–36, 50–51, 65–66 years) [27] from eight municipalities in the county of Norrbotten, and cohort 2 an age-stratified total population sample recruited in 1992 (*n* = 7735; 85% of invited; 20–21, 35–36, 50–51, 65–66 years) in the same municipalities. Cohorts 3 and 4 were random population samples from the entire county recruited in 1992 (cohort 3: *n* = 4851; 85% of invited; 20–69 years) [28] and 1996 (cohort 4: *n* = 7420; 85% of invited; 20–74 years) [29], while cohort 5 was a sample of subjects with an adult onset of asthma recruited in 1995–1999 (*n* = 309; 19–60 years) [5]. The first four cohorts were cross-sectional studies with the primary aim of studying prevalence and prevalence change. Random or stratified samples from these four cohorts were clinically examined during the years following recruitment, and all subjects who fulfilled the preset criteria for asthma in these clinical examinations were included in the asthma cohort. Cohort 5 was a case–control study including subjects fulfilling the criteria for adult-onset asthma [5]. In general, the participation rates in these cohorts were high, with no or only limited bias due to non-participation [26].

### Inclusion criteria at study entry in 1986–2001

The preset asthma criteria for inclusion in the asthma cohort depended on what types of clinical examinations the subjects underwent. In cohort 1, an evaluation by a physician at the time of examinations (year 1986) identified 398 subjects as having asthma or highly suspected asthma. In cohort 5, all 309 subjects had fulfilled the strict criteria for having an adult onset of asthma during the year preceding the examination [5]. Regarding cohorts 1–4, four preset inclusion criteria (A–D), based on data from structured interviews and clinical examinations including spirometry and tests of bronchial hyperreactivity for subsamples, are presented as follows:

(A) report of physician-diagnosed asthma or ever having had asthma

(B) wheeze with breathlessness without having a cold last 12 months (asthmatic wheeze) in combination with at least one of (1) attacks of shortness of breath (SOB) or wheeze in the past 12 months caused by at least three different triggering factors, or (2) asthma medication use in the past 12 months

(C) attacks of SOB or wheeze in the past 12 months in combination with FEV_1_ reversibility of both ≥ 12% and ≥ 200 ml

(D) attacks of SOB or wheeze in the past 12 months in combination with bronchial hyperresponsiveness measured through methacholine challenges [provocation concentration producing a 20% fall in FEV_1_ (PC_20_) ≤ 2 mg/ml according to a method developed by Malmberg and co-workers [30] and PC_20_ ≤ 8 mg/ml according to a rapid method developed within the OLIN studies [5]].

Thus, one included subject could fulfill one, several, or all of the A–D criteria. In total, 398 subjects from cohort 1 and the 309 subjects from cohort 5 were not classified according to the A–D criteria, but were included on the basis of the recent physician diagnosis at the clinical examination (cohort 1) and the specific criteria of adult-onset asthma (cohort 5) (Table 1).

**Table 1.** Number of subjects fulfilling the different asthma inclusion criteria in the five population-based cohorts.

	Cohort 1 (*n* = 544)	Cohort 2 (*n* = 858)	Cohort 3 (*n* = 164)	Cohort 4 (*n* = 180)	Cohort 5 (*n* = 309)	Total (*n* = 2055)
Met any of the preset A, B, C, or D asthma criteria:	146	858	164	180	0	1348
Of which met criterion:						
A	71	539	79	99	0	
B	85	647	113	113	0	
C	12	34	12	7	0	
D	0	0	44	65	0	
Physician diagnosis at examination of cohort 1:	398	0	0	0	0	398
Adult incident asthma in cohort 5:	0	0	0	0	309	309

Each subject could fulfill one or several of the preset A, B, C, or D asthma criteria: (A) report of physician-diagnosed asthma or ever having had asthma; (B) wheeze with breathlessness without having a cold in the past 12 months (asthmatic wheeze) in combination with at least one of: (1) attacks of shortness of breath (SOB) or wheeze in the past 12 months caused by at least three different triggering factors, or (2) asthma medication use in the past 12 months; (C) attacks of SOB or any wheeze in the past 12 months in combination with FEV_1_ reversibility of both ≥ 12% and ≥ 200 ml; and (D) attacks of SOB or any wheeze in the past 12 months in combination with positive methacholine challenge.

### Clinical examinations at study entry in 1986–2001

The clinical examinations at study entry in 1986–2001 included detailed structured interviews about respiratory symptoms and diseases, associated risk factors, and comorbid conditions, and measurements of height, weight, and dynamic spirometry (Mijnhardt Vicatest 5 dry volume spirometer) using internally and externally validated local reference values [31]. Tests of reversibility and bronchial hyperresponsiveness, and skin-prick tests were performed in subsamples.

### Clinical examinations at follow-up in 2012–2014

All subjects in the asthma cohort who were alive and still living in the county of Norrbotten (as recorded in the National Population Registry) were invited to a clinical follow-up in 2012–2014. Those who did not attend the follow-up examination despite several invitations were defined as non-participants. The examination included a detailed structured interview about respiratory symptoms and diseases, associated risk factors and comorbid conditions, occupation and educational level, measurements of height and weight, pre- and post-bronchodilator spirometry (Jaeger Masterscope pneumotach spirometer), skin-prick testing with 10 common airborne allergens in those aged ≤ 60 years, and blood sampling. Data from the asthma control test (ACT), Global Initiative for Asthma (GINA) classification, health-related quality of life measured by the eight-item Short-Form Health Survey (SF-8) questionnaire, and data regarding occupational exposures were also collected. Reasons for non-participation were recorded. Mortality dates were collected from the National Population Registry up until the date of invitation to the clinical examinations.

The follow-up time in years was defined as the time between study entry and death among deceased subjects, between study entry and invitation among those who had moved from the county or did not participate (non-participants), and between study entry and date of examination among participants in the clinical follow-up.

### Statistical analyses

In bivariate analyses, the chi-squared test was used to test for differences in proportions and the Student’s *t *test for differences in means. Tests for differences in means across more than two groups were performed by analysis of variance (ANOVA). A *p* value < 0.05 was considered statistically significant.

Poisson regressions (with robust errors) were performed to identify factors associated with (i) mortality and (ii) non-participation in the clinical follow-up in 2012–2014. Age (numeric), gender (women as reference), body mass index (BMI) categories (normal weight as reference), smoking habits (never-smoking as reference), and socioeconomic groups based on occupation (manual workers in service as reference) were considered as potential risk factors and included in the models. The follow-up time was included as an offset variable in the models. Furthermore, as a proxy for the time of study entry, i.e. the start of the follow-up period, all models were also adjusted for initial cohort (cohort 1–5 described earlier). The results are presented as relative risks (RRs) with 95% Wald confidence intervals (CIs) and *p* values. Pre-bronchodilator FEV_1_% of predicted was included in secondary versions of the models, and so were asthma medication use, ischemic heart disease, and FEV_1_/forced vital capacity (FVC) < lower limit of normal (LLN) [31], respectively. Overall, there were very few internal missing data on specific questions and measures. Subjects with missing data were included in the multivariate analyses with the missing data labeled ‘missing’, and the results of this variable are not presented.

### Sensitivity analyses

The main Poisson regression analyses of mortality were performed in several subgroups and these results are presented in Supplementary Table 1. The subgroups are based on cohort, year of birth, gender, follow-up time, age at asthma onset, smoking habits, and BMI. Also, the main Poisson regression analyses of non-participation were performed including subjects who declined to participate (*n* = 276) as non-participants only, compared to participants (*n* = 1006). Owing to signs of over-dispersion in some models, negative binomial regression with the dispersion parameter included in the models was used as an alternative regression approach. Also, the Poisson regression models were performed without including the follow-up time as an offset variable.

## Results

### Sample characteristics and participation in the follow-up

Figure 1 describes the identification of the asthma cohort in the clinical examinations of samples from the five cohorts during the study entry years 1986–2001. In total, 2055 subjects fulfilled the preset asthma criteria and were included in the asthma cohort. The numbers (*n*) of subjects from each cohort who fulfilled the different preset asthma criteria are presented in Table 1.

**Figure 2.**
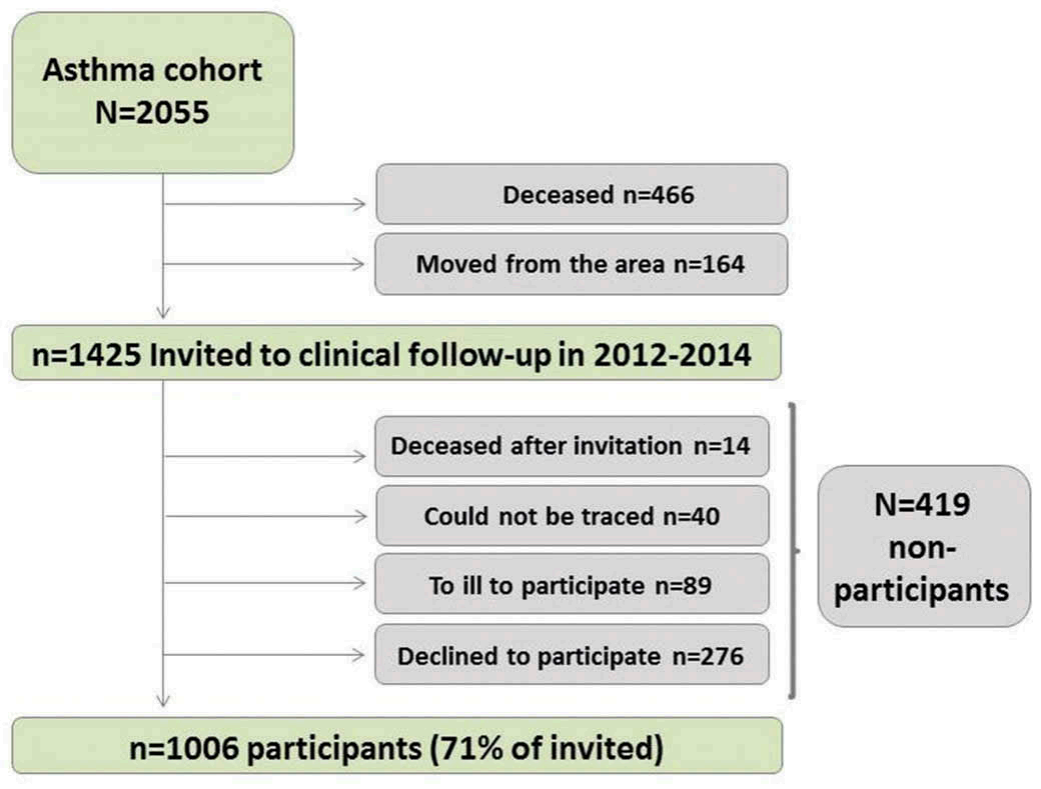
Participation and mortality in the asthma cohort at the follow-up in 2012–2014.

During the follow-up time, the cumulative mortality was 22.7% (*n* = 466) (Figure 2). At the time of invitation to the clinical follow-up, 1425 subjects were still alive and living in the county of Norrbotten. These were subsequently invited to the follow-up, in which 71% (*n* = 1006) participated while 29% (*n* = 419) did not (Figure 2). The mean (min–max) follow-up time was 18.7 (10–28) years among both participants and non-participants in the follow-up, which was similar to 18.5 (10–26) years among those who had moved from the county. The mean time (min–max) between study entry and death among the deceased was 14.2 (0.5–28) years.

The sample characteristics at study entry are presented in Table 2 (separate results for women and men are presented in Supplementary Table 2). The mean age among the 2055 subjects was 45.4 years and did not differ between men and women. About one-third of the asthma cohort (30.3%) was current smokers and 16.0% were obese at study entry. The participants (*n* = 1006) were younger than non-participants (*n* = 419) at study entry (40.5 vs 45.6 years; *p* < 0.001), and less predominantly female (55.4% vs 63.0%; *p* < 0.001). The mean age among male participants and non-participants was 40.6 and 42.9 years (*p* = 0.051), compared to 40.4 and 47.2 years (*p* < 0.001) among female participants and non-participants, respectively. Subjects who were alive but had moved from the county at the time of invitation to the follow-up (*n* = 164) had the lowest mean age (33.0 years) and were least often obese (11.6%). In contrast, the 466 deceased subjects were oldest at study entry (mean age 60.1 years) and also tended to be most frequently obese (18.7%). Also, the distribution of socioeconomic status differed between the subgroups (Table 2).

**Table 2.** Sample characteristics at study entry in 1986–2001 among all subjects and within different subgroups based on participation in the clinical follow-up in 2012–2014.

	Subgroup in clinical follow-up in 2012–2014					
	Participants	Invited non-participants	Had moved from county at time of invitation	Deceased at time of invitation		All subjects in the asthma cohort
Characteristic	*n* = 1006	*n* = 419	*n* = 164	*n* = 466	*p*	*n* = 2055
Female gender	55.4%	63.0%	54.3%	47.2%	< 0.001	55.0%
Mean age (years)	40.5	45.6	33.0	60.1	< 0.001	45.4
Original cohort						
Cohort 1	18.1%	21.7%	17.7%	51.9%	< 0.001	26.5%
Cohort 2	42.0%	44.6%	49.4%	36.1%	0.009	41.8%
Cohort 3	8.7%	8.4%	4.3%	7.5%	0.254	8.0%
Cohort 4	11.0%	11.5%	7.3%	2.4%	< 0.001	8.9%
Cohort 5	20.1%	13.8%	21.3%	2.1%	< 0.001	14.8%
Smoking habits						
Non-smoker	44.3%	42.2%	49.1%	28.2%	< 0.001	40.6%
Ex-smoker	28.2%	24.3%	23.3%	37.3%	< 0.001	29.1%
Current smoker	27.4%	33.4%	27.6%	34.5%	0.017	30.3%
Socioeconomic group						
Manual work in industry	17.7%	16.9%	10.4%	24.7%	< 0.001	18.5%
Manual work in service	28.9%	38.2%	27.4%	30.5%	0.004	31.0%
Assistant non-manual employees	16.1%	14.6%	15.2%	12.0%	0.234	14.8%
Intermediate non-manual employees	18.8%	12.2%	16.5%	7.3%	< 0.001	14.6%
Professionals and executives	4.1%	2.1%	5.5%	3.2%	0.166	3.6%
Self-employed non-professionals	3.9%	4.8%	3.0%	4.5%	0.741	4.1%
Students and homemakers	6.6%	6.4%	16.5%	3.9%	< 0.001	6.7%
Others^a^	4.0%	4.8%	5.5%	13.9%	< 0.001	6.5%
BMI group^b^						
Underweight	5.3%	5.2%	10.3%	2.8%	0.004	5.1%
Normal weight	46.1%	42.0%	49.0%	36.6%	0.004	43.4%
Overweight	34.5%	32.1%	28.4%	40.5%	0.016	34.8%
Obese	14.1%	20.7%	12.3%	20.1%	0.002	16.6%

*p* = Chi-square or ANOVA for tests of differences between subgroups, as appropriate.

*n* = 3 lacked data on smoking habits, *n* = 85 lacked data on body mass index (BMI).

^a^Data missing, unable to classify, or unemployed without report on previous occupation.

^b^Underweight = BMI < 20; normal weight = 20 ≤ BMI < 25; overweight = 25 ≤ BMI < 30; obese = BMI ≥ 30 kg/m^2^.

The prevalence of respiratory symptoms differed between subgroups, and was largest among those deceased by the time of follow-up (Table 3, with separate results for women and men presented in Supplementary Table 3). Among all 2055 subjects, 77.1% either had attacks of SOB or used asthma medicines, and 95.3% had any wheeze or attacks of SOB or used asthma medicines at study entry. When comparing participants with non-participants in the follow-up, 94.2% and 95.2% (*p* = 0.453), respectively, had any wheeze or attacks of SOB or used asthma medicines at study entry. The prevalence of allergic comorbid conditions was lowest while the prevalence of ischemic heart disease and FEV_1_/FVC < LLN was highest among those deceased by the time of follow-up, and these subjects also had the lowest mean values of both FEV_1_ and FVC at study entry (Table 3).

**Table 3.** Prevalence (%) of asthma-related characteristics, respiratory symptoms, comorbidities and lung function at study entry in 1986–2001 among all subjects and within different subgroups based on participation in the clinical follow-up in 2012–2014.

	Subgroup in clinical follow-up in 2012–2014					
	Participants	Invited non-participants	Had moved from county at time of invitation	Deceased at time of invitation		All subjects in the asthma cohort
Asthma-related characteristic	*n* = 1006	*n* = 419	*n* = 164	*n* = 466	*p*	*n* = 2055
Family history of asthma (%)	41.2	42.0	41.5	33.5	0.023	39.6
Any asthma medication in past 12 months (%)	36.6	37.9	36.0	48.9	< 0.001	39.6
Age (years) at asthma onset (%)						
Pre-school age	15.1	13.4	19.7	10.5	0.070	14.2
School age up to 15 years	13.7	12.3	15.6	6.2	0.004	12.0
16–30 years	25.0	26.4	32.8	10.2	< 0.001	22.8
> 30 years	46.2	47.8	32.0	73.1	< 0.001	51.1
Respiratory symptoms (%)						
Attacks of SOB	72.2	69.0	72.6	62.2	0.001	69.3
Any wheeze in past 12 months	88.9	88.1	87.2	95.1	< 0.001	90.0
Recurrent wheeze	78.0	78.3	74.4	77.9	0.755	77.8
Asthmatic wheeze	76.3	74.5	76.8	81.3	0.082	77.1
Persistent wheeze	23.9	25.1	25.0	44.2	< 0.001	28.8
Night-time sleep disturbance due to breathlessness or wheeze in past 12 months	40.2	43.7	40.2	60.7	< 0.001	45.5
Comorbid conditions (%)						
Rhinitis	53.5	50.8	56.7	45.9	0.026	51.5
Ever hayfever	43.3	38.7	53.0	25.3	< 0.001	39.1
Ever eczema	35.5	33.7	42.7	22.7	< 0.001	32.8
Ischemic heart disease	3.6	10.3	0.6	29.4	< 0.001	10.6
FEV_1_/FVC < LLN	8.3	10.1	7.5	25.1	< 0.001	12.4
Lung function (mean values)						
FEV_1_% of predicted	88.4	85.7	90.3	68.7	< 0.001	83.6
FVC% of predicted	87.0	84.8	88.1	70.8	< 0.001	83.0
FEV_1_/FVC	0.81	0.79	0.82	0.73	< 0.001	0.79
*Z* score FEV_1_	−1.05	−1.25	−0.90	−2.56	< 0.001	−1.42
*Z* score FVC	−1.12	−1.25	−1.07	−2.28	< 0.001	−1.40
*Z* score FEV_1_/FVC	0.21	0.13	0.40	−0.60	< 0.001	0.03
FEV_1_ reversibility (%)^a^						
< 12%	86.0	81.4	92.5	66.3	< 0.001	78.7
12–20%	10.0	11.4	5.7	18.7	0.007	13.0
20%	4.1	7.1	1.9	15.0	< 0.001	8.3

*p* = Chi-square test for difference between subgroups.

*n* = 790 women and 374 men lacked information on age at asthma onset (information on asthma onset was not included in the interview for cohort 1, and lacking for a few subjects in the other cohorts). *n* = 27 lacked adequate spirometry data.

^a^Reversibility testing results are only available from a subgroup of 710 subjects from cohorts 1 and 2: in cohort 1, subjects with forced expiratory volume in 1 sec (FEV_1_) < 85% were invited for reversibility testing (*n* = 273 participated); in cohort 2, subjects with FEV_1_ < 90% or FEV_1_/ vital capacity (VC) < 0.7 were invited for reversibility testing (*n* = 437 participated).

SOB, shortness of breath; LLN, lower limit of normal.

### Factors independently associated with mortality

Male gender, current smoking, and older age were significantly and independently associated with mortality (Table 4). The significance for ex-smoking was lost when FEV_1_% of predicted was included in the model, and decreased FEV_1_ was significantly associated with mortality. Neither any asthma medication use nor FEV_1_/FVC < LLN was a significant risk factor or changed any of the estimates for the other factors when included in the models, but ischemic heart disease was significantly associated with mortality. With manual workers in service as reference, self-employed non-professionals had a lower mortality risk.

**Table 4.** Risk factor analysis for mortality by Poisson regression, with results presented as risk ratio (RR) with 95% confidence interval (CI).

	Unadjusted		Model 1		Model 2		Model 3		Model 4		Model 5	
Covariate	RR	(95% CI)	RR	(95% CI)	RR	(95% CI)	RR	(95% CI)	RR	(95% CI)	RR	(95% CI)
Male gender	**1.37**	**(1.16–1.60)**	**1.40**	**(1.15–1.71)**	**1.40**	**(1.15–1.71)**	**1.40**	**(1.15–1.71)**	**1.38**	**(1.13–1.69)**	**1.40**	**(1.15–1.71)**
Smoking												
Current smoker	**1.64**	**(1.33–2.02)**	**1.84**	**(1.49–2.27)**	**1.57**	**(1.27–1.93)**	**1.57**	**(1.27–1.94)**	**1.56**	**(1.27–1.93)**	**1.57**	**(1.27–1.94)**
Ex-smoker	**1.84**	**(1.51–2.26)**	**1.34**	**(1.10–1.63)**	1.15	(0.94–1.40)	1.15	(0.94–1.40)	1.13	(0.92–1.37)	1.15	(0.94–1.40)
BMI^a^												
Underweight	0.64	(0.37–1.11)	0.86	(0.48–1.56)	0.77	(0.43–1.38)	0.77	(0.42–1.39)	0.78	(0.44–1.39)	0.77	(0.43–1.38)
Overweight	**1.38**	**(1.14–1.67)**	0.95	(0.80–1.14)	0.98	(0.82–1.18)	0.98	(0.82–1.18)	0.96	(0.80–1.16)	0.98	(0.82–1.18)
Obese	**1.44**	**(1.14–1.80)**	1.03	(0.82–1.30)	1.10	(0.87–1.38)	1.10	(0.87–1.38)	1.07	(0.85–1.36)	1.09	(0.86–1.38)
Socioeconomic group^b^												
Manual workers in industry	**1.36**	**(1.10–1.67)**	1.02	(0.79–1.32)	1.02	(0.80–1.32)	1.02	(0.80–1.32)	0.99	(0.77–1.28)	1.02	(0.80–1.32)
Assistant non-manual employees	0.83	(0.63–1.09)	0.79	(0.59–1.04)	0.83	(0.63–1.09)	0.83	(0.63–1.09)	0.80	(0.61–1.07)	0.83	(0.63–1.10)
Intermediate non-manual employees	**0.51**	**(0.36–0.72)**	0.71	(0.50–1.01)	0.74	(0.52–1.05)	0.74	(0.52–1.05)	0.73	(0.52–1.04)	0.74	(0.52–1.05)
Professionals and executives	0.91	(0.57–1.46)	0.89	(0.55–1.45)	0.79	(0.49–1.27)	0.79	(0.49–1.27)	0.78	(0.48–1.27)	0.79	(0.49–1.27)
Self-employed non-professionals	1.11	(0.75–1.65)	**0.61**	**(0.39–0.95)**	**0.60**	**(0.38–0.93)**	**0.60**	**(0.38–0.93)**	**0.58**	**(0.37–0.91)**	**0.60**	**(0.38–0.93)**
Students and homemakers	**0.59**	**(0.37–0.92)**	1.35	(0.90–2.04)	1.43	(0.94–2.18)	1.43	(0.94–2.19)	1.38	(0.92–2.08)	1.43	(0.93–2.18)
Others^c^	**2.18**	**(1.74–2.73)**	1.09	(0.84–1.41)	1.02	(0.79–1.31)	1.02	(0.79–1.31)	1.00	(0.77–1.29)	1.02	(0.79–1.32)
Age	**1.09**	**(1.08–1.09)**	**1.10**	**(1.09–1.11)**	**1.09**	**(1.08–1.10)**	**1.09**	**(1.08–1.10)**	**1.08**	**(1.07–1.09)**	**1.09**	**(1.08–1.10)**
FEV_1_% of predicted	**0.97**	**(0.96–0.97)**			**0.98**	**(0.98–0.99)**	**0.98**	**(0.98–0.99)**	**0.98**	**(0.98–0.99)**	**0.98**	**(0.98–0.99)**
Any asthma medication	**1.46**	**(1.25–1.71)**					1.01	(0.86–1.19)				
Ischemic heart disease	**3.53**	**(3.06–4.06)**							**1.43**	**(1.20–1.71)**		
FEV_1_/FVC < LLN	**2.37**	**(2.01–2.80)**									0.98	(0.79–1.22)

All covariates/factors were measured at study entry. All models were also adjusted for original cohort at study entry and with follow-up time included as an offset variable.

Model 1 includes 2019 subjects (21.3% mortality) with complete data, while models 2–5 include 1999 subjects (21.3% mortality) with complete data.

^a^Underweight = body mass index (BMI) < 20; normal weight = 20 ≤ BMI < 25 (reference category); overweight = 25 ≤ BMI < 30; obese = BMI ≥ 30 kg/m^2^.

^b^Manual workers in service (*n* = 638) is the reference category for socioeconomic group.

^c^Data missing, unable to classify, or unemployed without report on previous occupation.

Bold figures indicate statistical significance (*p* < 0.05).

FEV_1_, forced expiratory volume in 1 sec; FVC, forced vital capacity; LLN, lower limit of normal.

### Factors independently associated with non-participation

Older age and a history of ischemic heart disease at study entry were significantly and independently associated with non-participation in the 2012–2014 follow-up (Table 5). Neither gender, nor FEV_1_% of predicted, nor any asthma medication use was a significant risk factor or changed any of the estimates for the other factors when included in the models. Obesity was only borderline significant in adjusted analyses except for when FEV_1_/FVC < LLN was included in the model, and then obesity reached significance. With manual workers in service as reference, non-manual employees were participants to a larger extent.

**Table 5.** Risk factor analysis for non-participation (invited but did not participate) in the 2012–2014 follow-up by Poisson regression, with results presented as risk ratio (RR) with 95% confidence interval (CI).

	Unadjusted		Model 1		Model 2		Model 3		Model 4		Model 5	
Covariate	RR	(95% CI)	RR	(95% CI)	RR	(95% CI)	RR	(95% CI)	RR	(95% CI)	RR	(95% CI)
Male gender	**0.87**	**(0.67–0.94)**	0.87	(0.71–1.06)	0.88	(0.72–1.07)	0.87	(0.72–1.07)	0.87	(0.71–1.06)	0.87	(0.71–1.06)
Smoking												
Current smoker	1.18	(0.99–1.42)	1.14	(0.95–1.37)	1.13	(0.94–1.35)	1.06	(0.72–1.56)	1.07	(0.73–1.57)	1.12	(0.93–1.34)
Ex-smoker	0.93	(0.76–1.15)	0.85	(0.69–1.04)	0.84	(0.68–1.03)	0.94	(0.78–1.14)	0.95	(0.78–1.15)	0.84	(0.68–1.03)
BMI^a^												
Underweight	1.05	(0.72–1.54)	1.07	(0.73–1.58)	1.06	(0.72–1.56)	1.06	(0.72–1.56)	1.07	(0.73–1.57)	1.06	(0.72–1.56)
Overweight	1.02	(0.84–1.23)	0.94	(0.77–1.14)	0.94	(0.78–1.14)	0.94	(0.78–1.14)	0.95	(0.78–1.15)	0.94	(0.78–1.14)
Obese	**1.38**	**(1.12–1.71)**	1.23	(0.99–1.52)	1.23	(1.00–1.53)	1.23	(1.00–1.53)	1.22	(0.99–1.51)	**1.24**	**(1.01–1.54)**
Socioeconomic group^b^												
Manual workers in industry	0.80	(0.64–1.01)	0.92	(0.70–1.20)	0.90	(0.69–1.18)	0.90	(0.69–1.18)	0.90	(0.69–1.18)	0.90	(0.69–1.18)
Assistant non-manual employees	**0.77**	**(0.60–0.99)**	**0.78**	**(0.61–0.99)**	**0.76**	**(0.59–0.98)**	**0.76**	**(0.60–0.98)**	**0.76**	**(0.59–0.97)**	**0.76**	**(0.59–0.97)**
Intermediate non-manual employees	**0.60**	**(0.46–0.79)**	**0.65**	**(0.49–0.85)**	**0.63**	**(0.48–0.83)**	**0.63**	**(0.48–0.83)**	**0.63**	**(0.48–0.83)**	**0.63**	**(0.48–0.83)**
Professionals and executives	**0.51**	**(0.28–0.93)**	0.57	(0.30–1.05)	0.58	(0.31–1.07)	0.58	(0.31–1.07)	0.59	(0.32–1.09)	0.57	(0.31–1.06)
Self-employed non-professionals	0.96	(0.66–1.39)	0.92	(0.65–1.31)	0.92	(0.65–1.30)	0.92	(0.65–1.30)	0.91	(0.64–1.28)	0.91	(0.65–1.30)
Students and homemakers	0.82	(0.58–1.15)	1.00	(0.71–1.42)	1.00	(0.71–1.42)	1.00	(0.70–1.41)	1.00	(0.70–1.41)	1.00	(0.71–1.41)
Others^c^	0.94	(0.64–1.37)	0.96	(0.65–1.42)	0.94	(0.64–1.39)	0.94	(0.64–1.39)	0.94	(0.64–1.39)	0.94	(0.64–1.39)
Age	**1.02**	**(1.01–1.03)**	**1.02**	**(1.01–1.03)**	**1.02**	**(1.01–1.03)**	**1.02**	**(1.01–1.03)**	**1.02**	**(1.01–1.03)**	**1.02**	**(1.01–1.03)**
FEV_1_% of predicted	**0.99**	**(0.99–1.00)**			1.00	(0.99–1.00)	1.00	(0.99–1.00)	1.00	(0.99–1.00)	1.00	(0.99–1.00)
Any asthma medication	1.04	(0.88–1.23)					0.97	(0.81–1.15)				
Ischemic heart disease	**1.95**	**(1.56–2.43)**							**1.32**	**(1.03–1.69)**		
FEV_1_/FVC < LLN	1.16	(0.89–1.51)									1.14	(0.85–1.52)

All covariates/factors were measured at study entry. All models are also adjusted for original cohort at study entry and with follow-up time included as an offset variable.

Model 1 includes 1425 subjects (29.4% non-participants) with complete data, while models 2–5 include 1415 subjects (29.4% non-participants) with complete data.

^a^Underweight = body mass index (BMI) < 20; normal weight = 20 ≤ BMI < 25 (reference category); overweight = 25 ≤ BMI < 30; obese = BMI ≥ 30 kg/m^2^.

^b^Manual workers in service (*n* = 451) is the reference category for socioeconomic group.

^c^Data missing, unable to classify, or unemployed without report on previous occupation.

Bold figures indicate statistical significance (*p* < 0.05).

FEV_1_, forced expiratory volume in 1 sec; FVC, forced vital capacity; LLN, lower limit of normal.

### Sensitivity analyses

The sensitivity analyses in different subgroups are presented in Supplementary Table 1 and confirmed the main results of significant risk factors for mortality.

The main results for both mortality and non-participation were also similar in the negative binomial regressions with the dispersion parameter included in the models, and in the Poisson regressions performed without including the follow-up time as an offset variable.

## Discussion

This study provides a detailed characterization of a cohort identified in clinical examinations of population-based samples during 1986–2001. At the time-point of the examinations, 95% of the subjects experienced respiratory symptoms common in asthma and/or used asthma medicines. The main findings were that male gender, current smoking, older age, lower FEV_1_% of predicted, and ischemic heart disease at study entry were independent risk factors for mortality among adult subjects with asthma followed over 10–28 years. Furthermore, in this long-term follow-up, older ages, obesity, and ischemic heart disease were independently associated with non-participation. Lower socioeconomic status was associated with both mortality and non-participation in our study. These results provide an excellent platform on which to base further studies on persistence, remission, disease severity, and progress, including health-related quality of life and asthma control, and related factors. Longitudinal studies of adult asthma cohorts are warranted [8] and our study adds important knowledge to this field.

Within the European Community Respiratory Health Survey (ECRHS), both population-based samples and samples including subjects with asthma only have been studied longitudinally [32–35]. Among adults with asthma defined as a positive answer to either ‘Do you have or have you ever had asthma?’ or ‘Have you ever had asthma diagnosed by a doctor?’ aged 20–44 years at baseline in the RHINE study [32], the Nordic part of the ECRHS, 60% were females and 53% had allergic rhinitis, 63% had wheezing in the past 12 months, and 78% had any asthma symptom in the past 12 months at baseline. In our study, 95% had any wheeze or attacks of SOB or used asthma medicines, which implies more specific asthma criteria. Our current population-based asthma cohort was clinically examined at both study entry and follow-up, and can thus provide valuable results on asthma remission in future studies.

Several studies have compared all-cause [36–39] or cause-specific [36,38] mortality among subjects with and without asthma in population-based studies. These studies indicate that subjects with asthma have an increased risk of all-cause mortality which is related to the baseline FEV_1_ level [12,36,38,39], although the increased risk seems to be on the decrease [14,15,17]. However, independent risk factors for mortality among subjects with asthma are seldom presented in population-based studies or for well-characterized population-based asthma cohorts. In our study, we found that subjects with asthma had the same risk factors for mortality as most general population samples, such as male gender, older age, low FEV_1_, and smoking. One Danish study performed during 1974–1990, based on clinical examinations of patients with known or suspected asthma identified by general practitioners, indicated that baseline FEV_1_% of predicted, FEV_1_ reversibility, and smoking were independent risk factors for asthma-specific mortality among patients with asthma, both allergic and non-allergic [22]. In our study, lower socioeconomic status was also associated with mortality, a result in line with others [40]. Disease severity assessed by symptom burden and detailed data on asthma medicine use, as well as asthma control, may also be of importance for the risk of death from asthma, but more detailed analyses of our data set is required. However, in our study, low FEV_1_ was an independent factor related to mortality. As FEV_1_ may be a marker of disease severity we can speculate that having severe asthma is related to increased risk of mortality.

The participation rates in epidemiological studies have been declining over time, and more rapidly during the past few decades. Attempts to study whether and how the decreasing participation rates affect the results have been made in studies on respiratory epidemiology [25,26,41–45], and the results all seem to indicate that non-participants typically are male, smokers, and younger subjects [26,44,45]. It can be reasonably assumed that prevalence estimates may be more affected by increasing non-participation than associations between a risk factor and an outcome [25,26]. Studies on non-participation in long-term follow-ups of population-based asthma cohorts are rare, and our study indicates that non-participants are typically female, although non-significantly in adjusted analyses, and older, which contrasts with the results based on general population samples [26,44,45]. This could probably be due to the facts that subjects with asthma may develop a more severe disease and more comorbid conditions with increasing age [2,46], that women may be more affected by their disease or have a more persistent disease with poorer prognosis [7,10], and/or that the proposed obese female late-onset asthma phenotype is severe and more progressive [7,46,47], making these subjects less prone or able to participate. The association with low socioeconomic status is also of interest, and prior studies of general population samples have shown similar results and that full-time work is associated with non-participation [44].

There are some weaknesses with our study that should be mentioned. For one, tests of reversibility and bronchial hyperreactivity were not available for all subjects. The inclusion criteria permit that subjects who have intermittent remission or who have grown out of their asthma are included in the asthma cohort. However, 95% of the subjects reported respiratory symptoms common in asthma and/or asthma medication use at study entry, which suggests that only a few with remission of asthma were included in this adult asthma cohort. Also, factors measured at study entry such as asthma medication use may not be accurate predictors over a 10–28 year follow-up period owing to changes in, for example, available treatments and treatment guidelines and practices. The strength of our study is the large and well-characterized population-based asthma cohort followed for a long time. Spirometry was performed according to guidelines and was only lacking for 27 out of the 2055 included subjects, the methods included well-validated questionnaires, and the examinations and interviews were performed by well-trained staff. The participation rates were high, both in the clinical examinations in which the asthma cohort was identified and in the follow-up. Furthermore, Sweden has a complete population registry with reliable information on the date of mortality.

In conclusion, in this population-based adult asthma cohort the vast majority experienced respiratory symptoms common in asthma and/or used asthma medications at study entry. The risk factors associated with mortality were similar to those commonly identified in general population samples. Obesity, ischemic heart disease, low socioeconomic status, and older ages were associated with non-participation in the long-term follow-up. The detailed characterization of the cohort provides an excellent platform on which to base future studies on persistence, remission, disease severity, and related factors.

## Supplementary Material

ZECR_A_1334508_Supplementary_data.zip

## Geolocation information

The study area was the county of Norrbotten, Sweden.
